# Collection and use of human materials during TB clinical research; a review of practices

**DOI:** 10.1186/s12910-022-00776-x

**Published:** 2022-03-29

**Authors:** Joseph Ochieng, Betty Kwagala, Nelson Sewankambo

**Affiliations:** 1 Department of Anatomy, School of Biomedical Sciences, College of Health Sciences, Makerere University, P.O Box 7072, Kampala, Uganda; 2 School of Statistics, College of Business and Management, Makerere University, Kampala, Uganda; 3 School of Medicine, College of Health Sciences, Makerere University, Kampala, Uganda

**Keywords:** Collection, Storage, Use, Human biological materials, TB clinical research

## Abstract

**Background:**

Human biological materials are usually stored for possible future use in research because they preserve valuable biological information, save time and resources, which would have been spent on collection of fresh samples. However, use of these materials may pose ethical challenges such as unauthorized disclosure of genetic information, which can result in dire consequences for individuals or communities including discrimination, stigma, and psychological harm; has biosecurity implications; and loss of control or ownership of samples or data. To understand these problems better, we evaluated the extent to which tuberculosis (TB) clinical research protocols that were used to collect and store biological materials for future use conform to the requirements stated in the Uganda national guidelines for research involving humans as participants.

**Methods:**

This was a retrospective review of TB clinical research projects approved by the Uganda National Council for Science and Technology (UNCST) from 2011 to 2015, to examine whether they fulfilled the requirements for ethical collection and use of human materials. Data were abstracted through review of the project protocols using a template developed based on the informed consent and the Materials Transfer Agreement (MTA) requirements in the national guidelines.

**Results:**

Out of 55 research protocols reviewed, most of the protocols 83.6% had been used to collect the stored samples (sputum, blood and sometimes urine), 28% had a section on specimen collection and 24% mentioned ownership of the biological materials. With respect to review of the consent forms used in the studies that stored materials for future use, only 9% of the protocols had a separate consent form for storage of materials, 4.5% of the consent forms explained the risks, 11.4% explained the purpose of the study while 6.8% mentioned the place of storage for the collected materials.

**Conclusion:**

Many of the studies reviewed did not meet the requirements for collection and storage of biological materials contained in the national guidelines, which indicates a need to additional training on this topic.

## Background

Clinical research involves collection of human biological materials (HBMs) aimed at understanding the biology of the individuals in order to design appropriate interventions to address the health needs of the individuals or the wider community. With the exponential increase of collaborative research between the high and low -income countries (LMICs) particularly during the HIV/AIDS era, most of the (HBMs) collected during clinical research in these countries are exported to their richer counterparts for analysis and sometimes storage for future use [1, 2]. HBMs are often stored for possible uses in future research because they preserve valuable biological information, save time and resources which would have been spent on collection of fresh samples, and are less burdensome to sample sources [2–5]. Hence, it is becoming increasingly common to collect and store such materials for future potential unknown uses [6–10].

HBMs are often exported due to inadequate local scientific, technical and storage capacity, for purposes of quality assurance, by citizens of LMICs studying abroad, and because it is cheaper to analyze and interpret associated data in advanced facilities abroad.

Exportation of HBMs raises concerns on the part of LMICs regarding lack of control over the materials or data, place of storage, ownership of the materials and how they are used, for what and by whom and sharing benefits, if any [11–14]. This is mainly because use of stored human materials may negatively impact the sample sources, in case of disclosure of genetic information about an individual or community. Potential dire consequences of such disclosure include stigma, psychological harm, discrimination or biosecurity [4, 10, 15].

Over the years, Uganda has made significant progress in research ethics capacity development including enactment of the Uganda National Council for Science and Technology (UNCST) Law, establishment of national ethical research guidelines for conduct of research involving humans as research participants, institutionalization of the ethical review processes, and relevant human resource development (regulators at UNCST, researchers, ethical review committee members and their staff) [16]. In an endeavour to address ethical issues associated with use of stored human materials, the national ethical guidelines for research involving humans as participants were revised in 2007 to incorporate guidance on how human materials could be used ethically. This involved inclusion of a requirement for a separate informed consent form for storage and future use of human materials and a requirement for Materials Transfer Agreement (MTA) for exported materials [17].

Being one of the major causes of morbidity and mortality in Uganda, TB has been researched extensively. Therefore, this study examined how the TB clinical research protocols conformed to the Uganda national guidelines requirements for collection, storage and eventual use of stored human biological samples.

## Methods

This was a retrospective study of TB clinical research protocols approved and cleared by the UNCST from 2011 to 2015 to assess their compliance with the requirements for ethical collection and use of stored HBMs. The investigator reviewed TB research protocols in the presence and assistance of an authorized UNCST staff. Permission to review the protocols was granted by the UNCST after the investigator signed a confidentiality non-disclosure agreement. Data were abstracted using templates based on the informed consent and MTA frameworks in the Uganda national guidelines for conducting research involving humans as participants 2007. Additional data were collected on information describing materials collection and handling in the research protocols. Only clinical research protocols involving TB were reviewed by this study.

### Data sources and management

Data were independently captured by two data entry clerks into an EPIdata system. All the captured data were transferred into the Stata version 12.0, where each variable for every unique questionnaire was compared. This eliminated any data inconsistence as a result of entry. Other data inconsistencies were crosschecked with the source documents.

Data were assessed on the key contents on the consent and MTA based on the UNCST guidelines 2007 in three key domains (i) storage of HBMs, (ii) *exchange of HBMs* and (iii) *ownership and use of HBMs.* Data were obtained on a total of 46 protocols for *domain-i* that assessed inclusion of 13 items required for informed consent form for storage of HBMs. A total of 8 protocols generated data on domain-ii that assessed content on the exchange of HBMs using 21 items required for the material transfers agreements (MTA). For domain-iii, 46 protocols were assessed on five items indicating inclusion of information concerning storage of materials (acquisition of materials, storage and Future Use, ownership of samples, exchange/transfer of HBMs both from and to or within country for Research Purposes). All items were coded with the responses as; *1: yes included and clearly indicated, 2: yes included but not clearly indicated, and 3: not included at all*.

### Statistical analysis

Exploratory data analysis was conducted on all variables to enable identification of outliers, missing data and ascertain distribution. Descriptive statistics were generated providing percent/proportions for categorical data when the number of observations was at least 20. For the domain-ii “*exchange of HBMs*” where the number of protocols was only 8, only the number of observations under each coded responses was presented instead of the percent distribution.

### Ethics approval

Ethical review and approval was sought from the Makerere University School of Biomedical Sciences Higher Degrees Research and Ethics Committee Ref number SBS385. Clearance to conduct the study was obtained from the Uganda National Council for Science and Technology (UNCST) Ref. number SS4165. The requirement for informed consent was waived since it was a retrospective review of research protocols achieved the UNCST the National Research Regulatory Agency with no individual human participants involved. A confidentiality agreement between the investigator and the UNCST was signed before access to research records could be granted. All methods were carried out in accordance with relevant guidelines and regulations. No individual protocol identifying information was recorded.

## Results

A total of 55 research protocols met the inclusion criteria and were reviewed. Most of the protocols 83.6% entailed collection of HBMs including sputum, blood, biopsies and sometimes urine; 28% had a section on specimen collection and 24% mentioned ownership of the HBMs (Table 1).

**Table 1:** Information in the protocol about collection and use of human materials

Items	Clearly included	Included but not clear	Not available
Material acquisition	28.3	47.8	23.9
Storage and future use	13.0	26.1	60.9
Ownership of materials	0.0	24.4	75.6
Exchange/transfer of materials	0.0	20.0	80.0
Exchange/transfer while abroad	0.0	13.3	86.7

Percentage of protocols with available information on materials collection

Although many of the studies store HBMs for future use, only 9% of the protocols had a separate consent form for storage of HBMs, 4.5% of the consent forms explained the risks associated with storage and use, 11.4% explained the purpose of storage, and 6.8% mentioned the place of storage for the collected HBMs (see Table 2).

**Table 2:** Review of the informed consent forms for storage and future use of human materials

Items (n = 46)	Clearly included	Included but not clear	Not available
Storage & enrol separated	9.1	11.4	79.5
Storage purpose	11.4	9.1	79.5
Storage quantity	2.3	2.3	95.5
Storage place	6.8	4.5	88.6
Confidentiality measures	2.3	6.8	90.9
Sample use governance	0.0	2.3	97.7
Storage risk/benefits	4.5	0.0	95.5
Other inform included	0.0	4.5	95.5
Storage future use	0.0	4.7	95.3
Ugandan Co-PI	0.0	0.0	100.0
No storage penalty	4.5	0.0	95.5
Storage withdrawal	6.8	0.0	93.2
REC to review future	0.0	4.5	95.5

Percentage of protocols with adequate Information in the approved consent forms

**Fig. 1:**
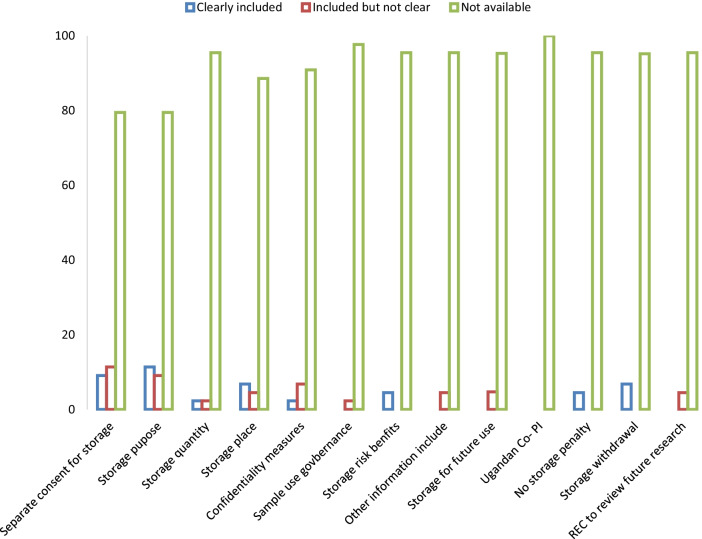
Adequacy of the informed consent forms

An overwhelming majority of the consent forms for storage and future use of human biological materials in TB clinical research studies were grossly inadequate when compared to the requirements in the national guidelines (Fig. 1).

Only 8 protocols had their MTAs accessed. Of the accessed MTAs, half (4/8) had a description of the HBMs to be transferred, 5 had the purpose for transfer and usage of HBMs, 4 had the names of the individuals covered by the agreements while another 4 had information describing the ownership of the HBMs (see Table 3).

**Table 3:** Review of the materials transfer agreements

Items	Clearly included	Included but not clear	Not available
MTA	8	0	0
Parties involved	7	1	0
Description of materials	4	2	2
Purpose and usage	5	2	1
Users names	4	2	1
Period of use	7	0	1
Description of disposal	4	1	3
Restrictions on usage	2	2	4
Ownership of derivatives	4	2	2
Information on ownership	4	3	1

Results following assessment of adequacy of the approved materials transfer agreements

## Discussion

The study set out to examine the extent to which TB clinical research protocols conform to the requirements of the Uganda national guidelines and found that most of the protocols are non-compliant.

Concerning HBMs collection, the majority of protocols lacked an elaborate section on collection, storage and use. This means that the HBMs collection procedures were not well documented in the protocols. Undocumented collection and handling of HBMs can affect the standards concerning their collection and handling, leaving room for abuse and violation of ethical standards by researchers. HBMs collection and use should be part of study procedures in any research protocol intending to collect HBMs and, such procedures are subject to ethical review and approval by the relevant research regulatory agencies before they can be carried out. Non-compliance with the national guideline’s requirements had been reported in earlier work, which reviewed research site monitoring reports in the country [18]. However, all the protocols reviewed had been approved by the Research Ethics Committee (REC) and cleared by the national regulator, UNCST. This means that both the researchers, the RECs and the UNCST failed to correctly implement the guidelines.

Although significant capacity in research ethics and associated research regulation has been built in Uganda, the quality of ethical review still needs improvement and this should be a continuous process. The challenge of low quality of ethical review in this setting has been observed in other related work [16]. Therefore, there is a need for retraining of all the concerned parties on the requirements by the guidelines.

The requirement for a separate informed consent form for storage of HBMs came in existence following the launching of the revised national guidelines in 2007. This was in response to whether participants who consent for storage of biological materials as part of the enrolment consent actually understood the consent process and appreciated the meaning and implications of their decisions. This study analysed studies approved and conducted at least four years (2007–2011) after revising the guidelines. It’s expected that four years would be sufficient to allow appreciation and adoption of the revised guidelines. However, the results of this study indicate that implementation of the guideline requirements was not adhered to, indicating either a lack of knowledge or understanding or disregard of the requirements of the guidelines on the part of both the researchers and the regulators. It should be noted that the presence of the guidelines alone may not necessarily translate in ethical conduct of research as observed in previous literature [19]. Additionally, it has been observed that although RECs globally have a mandate to protect research participant interests for instance, with respect to confidentiality, ownership, export, storage and secondary use of HBMs (individual good) with specific consent, regulations and policies, implementation of the requirement varied among the RECs [20, 21]. An additional challenge in the African context relates to traditional perceptions of blood and the body. It is well-documented that blood carries symbolic value for many Africans. Since blood has been associated with strength, exploitative relations, colonialism, and superstition relating to witchcraft, amongst others, it is important to carefully explain the purposes of collection and storage of blood samples in the consent process and during collection [22–27]. Similarly, other commentators have stated that Africans believe that blood obtained ostensibly for research purposes could be used for sorcery [28, 29].

Although it is a requirement that all HBMs exchanged by researchers from one institution to another must be accompanied by a valid materials transfer agreement (MTA), this was not the case with many of the studies reviewed. In order to export HBMs for research purposes from Uganda to other countries, it is a requirement to have a valid MTA, and an export permit must be sought from the UNCST. No records of such permits were identified during this records review. It was difficult to confirm if the studies reviewed had MTAs and export permits because this was a retrospective review of records which may miss out some of the documentation due to filing or storage issues.

Additionally, the accessed MTAs lacked the required information for a valid agreement as stipulated in the national guidelines (see Table 3). The findings of this study are indicative of a general problem associated with the low quality of ethical review and oversight for research conducted in the country as has been highlighted in previous literature [16]. Mandatory periodical context specific research ethics training for all research regulators, researchers and ethics review committee members that address collection and storage of HBM is essential. The training should focus on the requirements of the national guidelines. The training should precede clearance and data collection. Such requirements have been implemented for other purposes like Good Clinical Practice (GCP) training for clinical research.

There is need for continuing training of the ethics committees, researchers, and the research regulators on all the ethical requirements as stipulated in the national guidelines as well as updating on the changes affecting research ethics. Hence it should be a requirement that every research regulator and researcher should undergo research ethics training with focus on the national guidelines before getting involved in their activities.

## Limitations

Being a retrospective review, it is possible that some of the TB research protocols were not retrieved because of challenges for achieving and retrieval of study protocols and associated documents.

It was not possible to observe the actual processes of what took place because the review occurred after most of the studies had been completed. A more robust study with a prospective design would provide a comprehensive evaluation on the magnitude of the problem.

The findings of this study may not help to improve the quality of the studies that were analysed, being retrospective, but could be used to inform future research.

The investigator abstracted the data from the study protocols. Using two or more data collectors to abstract the data would have further validated the findings and strengthen the conclusions reached.

## Conclusion

Ethical requirements by the national guidelines for collection, storage and use of HBMs were hardly observed by the researchers, ethics committees, and research regulators in many approved TB clinical research studies.

## Data Availability

The data-sets used and/or analysed during the current study are available from the corresponding author on reasonable request.

## Abbreviations

HBM: Human biological materials
LMIC: Low and middle income countries
MTA: Materials transfer agreement
REC: Research Ethics Committee
TB: Tuberculosis
UNCST: Uganda National Council for Science and Technology
